# Cap-Independent Circular mRNA Translation Efficiency

**DOI:** 10.3390/vaccines11020238

**Published:** 2023-01-20

**Authors:** Andrei A. Deviatkin, Ruslan A. Simonov, Kseniya A. Trutneva, Anna A. Maznina, Anastasiia B. Soroka, Anna A. Kogan, Sofya G. Feoktistova, Elena M. Khavina, Olga N. Mityaeva, Pavel Y. Volchkov

**Affiliations:** 1 Life Sciences Research Center, Moscow Institute of Physics and Technology, National Research University, 141700 Dolgoprudniy, Russia; 2 Endocrinology Research Centre, 117036 Moscow, Russia; 3 Martsinovsky Institute of Medical Parasitology, Tropical and Vector-Borne Diseases, First Moscow State Medical University (Sechenov University), 119991 Moscow, Russia; 4 Faculty of Biology, Technion—Israel Institute of Technology, Haifa 32000, Israel

**Keywords:** circRNA, cap-independent translation, mRNA vaccines

## Abstract

Recently, the mRNA platform has become the method of choice in vaccine development to find new ways to fight infectious diseases. However, this approach has shortcomings, namely that mRNA vaccines require special storage conditions, which makes them less accessible. This instability is due to the fact that the five-prime and three-prime ends of the mRNA are a substrate for the ubiquitous exoribonucleases. To address the problem, circular mRNAs have been proposed for transgene delivery as they lack these ends. Notably, circular RNAs do not have a capped five-prime end, which makes it impossible to initiate translation canonically. In this review, we summarize the current knowledge on cap-independent translation initiation methods and discuss which approaches might be most effective in developing vaccines and other biotechnological products based on circular mRNAs.

## 1. Introduction

The more frequent and severe the infection, the more resources are invested into research on the interaction of the pathogen with the human organism. As a result, there is a need to develop effective approaches to treat and prevent disease. The most effective and safest method to prevent the spread of an infectious disease is vaccination. At the beginning of the COVID-19 pandemic, many types of vaccines were effectively used for controlling various diseases [1]. The development of alternative approaches was hampered to some extent, as there did not appear to be a problem that needed solving.

**Figure 1:**
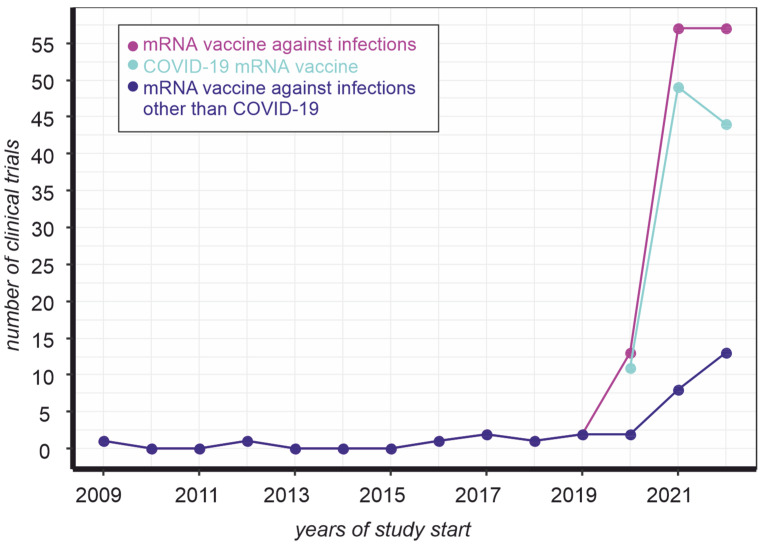
Dynamics of mRNA vaccine clinical trials registered on clinicaltrials.gov. The number of trials was counted by keyword ([condition “Infections”, title “mrna vaccine”] (magenta), [condition “Infections”, other terms “NOT coronavirus NOT COVID19 NOT BNT162b2”, title “mrna vaccine”] (blue), [condition “Infections”, other terms “coronavirus OR COVID19 OR BNT162b2”, title “mrna vaccine”] (cyan)) occurrence, as of 28 November 2022.

The emergence of a new challenge became a turning point in vaccine development. Indeed, there were virtually no clinical trials of mRNA vaccines to prevent infections before the COVID-19 pandemic (Figure 1, magenta line). At the same time, the number of mRNA-based vaccine trials against COVID-19 in the eleven months of 2022 does not exceed the same indicator for 2021 (Figure 1, cyan line). In contrast, the development of such drugs to prevent other infections almost doubled in one year (Figure 1, blue line). In other words, the mRNA vaccine platform that has been promoted to combat COVID-19 is increasingly being used as an approach to prevent infections in general.

However, mRNA technology has some weaknesses. Firstly, widely used mRNA vaccines do not maintain their stability at room temperature for more than 24 h [2]. Refrigeration at +4 degrees Celsius extends the shelf life of the vaccine by several weeks, while −20 degrees Celsius extends it by several months. Therefore, there is a clear need for a second-generation mRNA-based platform for vaccine development with increased stability to ensure adequate protection against future pandemics [2].

Secondly, the half-life of mRNA in the mammalian cell is estimated to be several minutes to several days, with a median value of 7 h [3]. Surprisingly, to our knowledge, the half-life of Moderna and Pfizer/BioNTech mRNA vaccines in human cells is unclear. This parameter influences the vaccine dose required to elicit a sufficient immune response.

The main reason for the instability of mRNA is its sensitivity to RNases. Exoribonucleases actively degrade the 5′ and 3′ ends of mRNA and play an important role in mRNA degradation [4]. Circular RNAs (circRNA) do not have 5′ and 3′ ends, which increases their stability compared to the linear form, which is one of its advantages. They are generally stable in serum, with a 24-h incubation at room temperature having minimal effect on titer [5]. In vivo, circRNA can remain in the cell for more than 48 h as they are resistant to degradation by exonucleases and localize mainly in the cytoplasm [6]. As such, they are a promising alternative to linear RNA for use in vaccines, as they do not require special equipment for storage. At the same time, to the best of our knowledge, currently, there are no registered clinical trials of vaccines against infectious diseases based on the circular mRNA platform.

There are several ways to synthesize circRNAs. These molecules can be produced directly in cells by transcription and back-splicing when delivered as vector DNA (e.g., AAV [7]). On the other hand, circRNAs can be synthesized in vitro [8,9]. Currently, there are three methods for circRNA vaccines in in vitro production: chemical-, enzyme-, and ribozyme-based ligations. At the same time, these approaches have shortcomings (reviewed in [10]). Therefore, new protocols for large-scale production are needed to enable the cost-effective design and production of circRNA vaccines.

**Figure 2:**
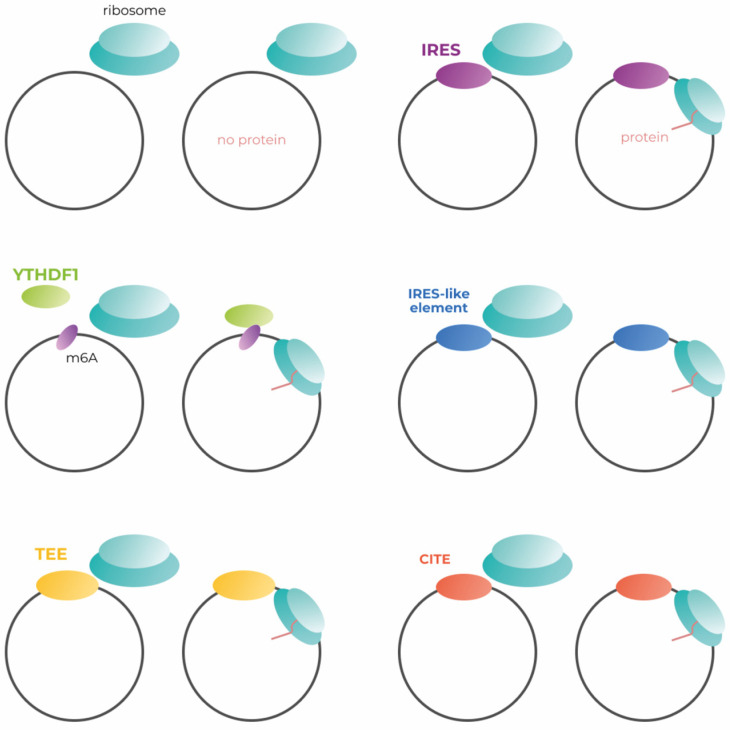
Simplified scheme of the mechanisms for cap-independent initiation of circRNA translation. IRES—internal ribosome entry site; m6A—N6-methyladenosine; YTHDC1—the reader of m6A modification; TEE—translation enhancing element; CITE—cap-independent translation enhancer.

Much of genetic construction is tailored to canonical translation, which begins with cap-dependent initiation. Endless circular RNAs have no cap necessary for canonical translation initiation (Figure 2, left upper panel). As far as we know, only one of the possible methods of cap-independent translation initiation is used in the development of modern circular mRNA vaccines, although there are several approaches to increase the effectiveness of transgene production. Here, we consider all known methods of translation initiation applicable to the engineering of circular mRNAs (Figure 2). In addition, we suggest which approaches might be most effective in the engineering of vaccines based on circular mRNAs.

## 2. Translation Initiation Mechanisms

**Figure 3:**
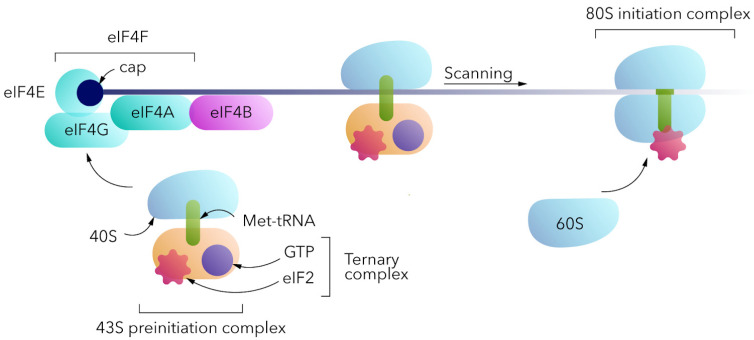
A simplified scheme of cap-dependent translation initiation mechanism.

For eukaryotic translation (Figure 3), the 80S ribosome should be assembled at the mRNA. The canonical cap-dependent process begins with the interaction of a small 40S ribosomal subunit with a ternary complex consisting of trimeric eukaryotic initiation factor 2 (eIF2), GTP and initiator transport RNA (Met-tRNAi) to form the 43S pre-initiation complex (PIC) [11,12]. 43S PIC binds to the 5′ end of the mRNA, interacting with the eIF4F complex (cap-binding eIF4E, the scaffolding eIF4G, and the helicase eIF4A). It should be noted that in the absence of the cap, the above complex does not bind to the 5′ end of the mRNA and translation would not be initiated in this way. 43S PIC and eIF4F complexes, together with poly(A)-binding proteins (PABPs) at the poly(A) tail, form the 48S PIC, which then scans a 5′-untranslated region in the 5′-3′ direction for the AUG start codon within the Kozak sequence context. Start codon recognition by methionine anticodon triggers GTP hydrolysis and eIF2-GDP release, as well as 60S ribosomal subunit interaction mediated by eIF5B to form the 80S initiation complex [13]. eIF1, eIF1A, eIF2, eIF3, eIF4B, eIF4F, eIF5, eIF5B, and PABP are the key factors involved in cap-dependent translation initiation.

### 2.1. Efficiency of IRES-Dependent Translation Initiation

The 5′UTR of mRNA has the potential to control translation independently of the cap via an internal ribosome entry site (IRES). Remarkably, cap-independent translation initiation can also be caused by other factors, which are discussed in the following sections. IRESs are present in both viral and cellular mRNAs. Cellular IRESs are very diverse, which makes them difficult to classify. In other words, there appear to be many different, as of yet uncharacterized, mechanisms that control cap-independent translation using endogenous IRESs [14].

Based on the differences in the way viral IRESs interact with ribosomes, they can be classified into four groups [15]. Group 1 IRESs bind to the ribosome without accessory factors. Group 2 IRESs bind to the ribosome with the help of eIFs and Met-tRNA. Group 3 IRESs bind to the ribosome using eIFs, Met-tRNA, as well as IRES transactivating factors (ITAFs), and function in rabbit reticulocyte lysate (RRL) without extracts from other cell types. Group 4 IRESs bind to the ribosome using eIFs, Met-tRNA, as well as ITAFs, and function in RRL only after addition of extracts from other cell types.

Originally, IRESs were studied as part of the viral replication system. Initial comparative studies showed low efficiency of IRES-associated translation initiation compared to cap-dependent initiation. In these studies, a bicitron system was used in which the first protein was synthesized in a classical cap-dependent manner and the second protein was translated by IRES. The efficiency of translation of the second protein was significantly lower [16,17]. The low protein expression induced by IRES was explained by the inefficient re-initiation of translation at an internal AUG codon downstream of IRES. IRES therefore gained a reputation as a less efficient translation initiation method as early as the 1980s, prompting researchers to modify IRES to improve the efficiency of translation initiation.

In the case of bicistronic cassettes, the efficiency of IRES-dependent translation initiation can be improved by a tailored intercistronic sequence [18]. The size of the intercistronic spacer sequence in the 5′ region of the IRES sequence has a significant impact on downstream protein expression. By choosing the spacer size, it was possible to achieve even stronger expression of proteins whose initiation was dependent on IRES and not on the cap. Another comparative study showed that IRES did not perform worse than cap under certain conditions [19]. Translation efficiency using EMCV IRES was similar compared to cap-dependent gene expression in the Lcl1D cell line, although efficiency remained low in many other cases [19]. This study compared the transcriptional efficiency of a gene in a bicistronic vector in which the initiation of the first protein was cap-dependent and that of the second protein was initiated by an EMCV IRES (type 2 IRES). Furthermore, it was shown that the efficiency of IRES strongly depends on the expressing cell line [19].

For more efficient use of IRES, it is worth choosing the correct combination of IRES type and express cell line. For example, group 1 IRESs (from PV, ECHO and HRV viruses) showed high translational efficiency in the HeLa, HepG2 and FRhK4 cell lines, while they were extremely inefficient in the BHK21 and Neuro-2A non-primate cell lines and in the SKNBE human neuronal cell line. At the same time, group 2 IRESs (from EMCV, FMDV and HCV) appear to be excellent candidates for the development of expression vectors, as they functioned efficiently in all cell lines tested by the authors [20].

Currently, there are several examples of successful developments based on IRES-mediated translation initiation. A circular mRNA prototype encoding the modified spike protein of SARS-CoV-2 induced neutralizing antibody titers for up to 7 weeks after secondary inoculation and had broad neutralizing activity against different viral strains [21]. In another study, circRNA was found to confer higher and more durable efficiency than analogous mRNA. The induced immunity persisted for at least 8 weeks after secondary inoculation [22].

In summary, the different IRES are differentially efficient in numerous cell types (Table 1). These data suggest that the proper selection of the most appropriate IRES type could provide an efficient circular mRNA platform. It makes more sense to consider the cell type in which the IRES-mediated translation initiation will occur. The cell type that is most similar to the target tissue is best suited for preclinical studies. Ideally, this should be a primary culture in which circular mRNAs are tested with as many IRES variants as possible. The most efficient variant should be selected for further experiments. In addition, circRNA reporter systems can be used as a suitable platform to test the efficiency of translation initiation of IRES-like elements [23].

### 2.2. m6A and Translation Initiation

The most common internal chemical RNA modification, N6-methyladenosine (m6A), is involved in the initiation of translation [28]. Even a single m6A modification within the 5′UTR of mRNA has the potential to recruit a 40S ribosomal subunit through direct eIF3 binding. Ribonucleotide methylation is a flexible process that depends on the activity of epitranscriptional “writer” and “eraser” factors [29].

The “readers” of the m6A modification bind mainly to a modified nucleotide in the RR(m6A)CH motif (R stands for purines, H for pyrimidines and C for the cytosine base). There are several representatives of the YTH domain-containing protein family: YTHDF1/2/3, localized in the cytoplasm of cells, and YTHDC1/2/3, localized in the nucleus [30].

Recently, a firefly luciferase assay was used to demonstrate that m6A-mediated translation initiation is less efficient compared to cap-dependent initiation [31]. On the other hand, this approach demonstrated comparable results in comparison to IRES-mediated translation initiation [32]. This technique can be applied in circular mRNA engineering, but the efficiency of such constructs should be carefully assessed.

### 2.3. Endogenous IRES-like Elements in Eukaryotic Genome

Several endogenous circRNAs are known to translate proteins in eukaryotic cells. Their 5′UTRs contain IRES-like sequences that drive translation independently of cap [33]. Fan et al. [23] found 97 short random hexamer sequences enriched in endogenous translating circRNAs (compared to linear mRNAs) that drive translation independently of cap. These IRES-like structures interact with various RNA-binding factors that initiate cap-independent protein expression. At the same time, RBPs are differentially expressed in various tissues [34]. This means that IRES-like sequences have different efficiencies in different tissues, which should be taken into consideration during implementation of IRES-like structures into genetic constructs. Mutation of these sequences significantly reduced the translation rate. In addition, some hexamers contain specific context-dependent m6A modifications of such RNAs, further complicating the system. Remarkably, virtually every random sequence longer than 50 nucleotides contains such a short IRES-like element. Indeed, the authors demonstrated that around 2% of all hexamers (97 out of 4096) may initiate translation. A sequence of 50 nucleotides can be divided into 45 possible hexamers, while a sequence of 100 nucleotides can be divided into 95 hexamers. Assuming that nucleotide sequences have some degree of randomness, we can conclude that any RNA sequence of any length can have translational potential if they contain an ORF. However, the identified elements showed great similarity with previously described IRES-like sequences of some endogenous genes, such as Hsp70 [35] and Gtx [36].

In addition, screenings have shown that almost 10% of eukaryotic mRNAs contain IRES-like sequences that can drive translation independently of the cap [37,38]. To date, it is not clear how effective such short sequences are in initiating translation compared to viral IRESs that have been previously used. At the same time, a comprehensive paper [23], published only half a year ago, showed that the efficiency of translation initiated by some hexamers is at least as good as initiation using the m6A modification.

### 2.4. Translation Enhancing Elements

Translation enhancing elements, TEE, are genomic sequences that facilitate cap-independent translation. They were first identified using a method based on the mRNA display principle [39]. In this study, a genomic library consisting of approximately 10^13^ randomly selected 150-nucleotide fragments of total genomic DNA was created. These sequences were placed in the 5′-UTR of the starter DNA construct encoding a His6 affinity tag.

After in vitro transcription, the resulting set of uncapped single-stranded RNAs was conjugated by photoligation with a puromycin residue at the 3′-end and subjected to translation. Remarkably, during translation, a chemical bond was formed between the newly translated peptides and their coding mRNA by the natural peptidyl transferase activity of the ribosome, which recognized puromycin as a tyrosyl-tRNA analogue. As a result, molecules capable of cap-independent translation consisted of uncapped mRNAs linked to His-tags. In addition, these substances were selected by immobilized metal ion affinity chromatography. The functional RNAs were then isolated, reverse transcribed and amplified by PCR to create the novel pool of DNA for another selection cycle. This procedure was repeated six times until the sequences capable of cap-independent translation enhancement became predominant in the library. This approach revealed more than 12 thousand human genome elements mediating translation in a cap-independent manner in vitro and in mammalian cells.

Several TEEs have shown extremely high efficiency in protein production. For example, the addition of hTEE-658 to the capped construct increased the expression of the target protein 100-fold compared to a construct without TEE [40]. According to a further study, this 37-nucleotide motif placed upstream of the certain protein coding region of transfection plasmid was capable of increasing the protein expression level in experiments with transfection and subsequent infection using the vaccinia virus (VACV) [41]. Notably, neither the length of the 5′-leader sequence, nor the distance between the motif and the coding region affects the ability of hTEE-658 to increase transcription and translation rates. To the best of our knowledge, the mechanism of TEE cap-dependent translation enhancement or cap-independent translation initiation is unclear.

### 2.5. Cap-Independent Translation Enhancers

Another method of cap-independent translation, based on cap-independent translation enhancers (CITEs), was first identified in the 3′UTR of plant viruses. This is thought to be activated under unfavorable conditions, such as during viral infection or tumor hypoxia, as cap-dependent mRNA translation in human cells is suppressed during these times [42]. In addition, previous research has shown that CITE-initiated translation of mRNAs can occur in human cell lines when these elements from plant viruses have been inserted into mRNAs [43].

In general, CITEs facilitate translation by binding to the eIF4E and/or eIF4G subunits of the translation initiation factor eIF4F, followed by attraction of the 40S ribosomal subunits. Importantly, some CITEs can bind directly to ribosomal subunits or the ribosomes themselves without being dependent on elF4F. In CITE-dependent translation, ribosomes are attracted to an internal element that binds eIF4G, but only enters the mRNA via the 5′ terminus, forming a hairpin [43]. In addition, 3′ CITEs can enhance translation of complementary sequences by facilitating long-distance interactions between the 3′CITE hairpins and the 5′ regions of subgenomic RNA. The translation efficiency of CITEs is variable and depends on the sequence being translated [44] as well as their type (5′ or 3′).

It was shown that 5′UTR-driven cap-independent translation is less efficient than cap-dependent initiation [42]. Conversely, there are a number of studies indicating that 3′CITEs are a highly efficient substitute for 5′-capped translation initiation [43,44,45]. Summarizing, it can be speculated that they represent an effective method of cap-independent translation and have the potential to be used as initiators of translation in circular mRNA vaccines.

### 2.6. R2 Elements

The human genome contains retrotransposon sequences. Their replication cycles consist of genomic DNA transcription, translation and reverse transcription of synthesized RNA [46]. At the same time, there is a group of retrotransposons with non-long terminal repeats that can be integrated into ribosomal genes [47]. As a result, transcription of these genomic regions occurs with the help of RNA polymerase I, which lacks the C-terminal domain where the capping proteins are located. This leads to the production of mRNAs without caps.

The best characterized representative of such retrotransposons, the R2 element, was found in insects, arthropods, nematodes, birds, and fish [48]. The 5′ of the untranslated region of R2 contains pseudoknots. It is thought that these conservative structures of the R2 element are recognized by translational machinery and can initiate translation [49]. Ruminski et al. have shown that the efficiency of such R2-mediated translation strongly depends on its sequence and likely on its structure. Moreover, some of the R2 elements demonstrated the translation of the luciferase gene to be more than 35 times more effective than that of HCV IRES [49]. In the context of circular mRNA, this means that the library of sequences with different pseudoknots should be tested to find the most efficient one to use for production.

## 3. Circular RNA as Delivery Vehicle for Protein Synthesis

Circular mRNA can be translated without special initiation sites such as IRES or m6A. Abe et al. have shown that circRNA encoding FLAG peptides can be translated without 5′-cap, poly(A), IRES, and even without a stop codon, carrying only the Kozak sequence [50]. The mechanism of this translation pathway is reminiscent of the mechanism of circular DNA replication of the “rolling circle” type, in which the enzyme reads the template code while “running” in a circle. As a result, the product of rolling circle translation is an infinite polyprotein consisting of repeating FLAG peptides. This approach can be optimized to produce more protein from the circRNA by adding an autocatalytic peptide instead of a stop codon. In this case, the translated polyprotein will “cut” itself into individual protein products. However, the concrete mechanism of translation initiation was not the focus of this pioneering work.

The possibility of producing protein from circular mRNAs with infinite ORF in a CHO cell line was demonstrated in 2019 by Costello et al. [51]. Three types of circular RNA were used in the experiments: (1) usual circular mRNA (C mRNA); (2) continuously translating circular mRNA (CTC mRNA) without a stop codon, which theoretically allows for the infinite translation of the protein; (3) 2A self-cleaving, continuously translating circular mRNA (2ACTC mRNA) without a stop codon. In the last construction, a 2A self-cleaving peptide sequence was added to allow co-translational cleavage of the growing polyprotein, which could theoretically yield an infinite number of similar proteins. Linear mRNA was used as a control and human erythropoietin (EPO) was chosen as a model protein. A significant increase in the amount of secreted EPO was demonstrated for the CTC mRNA and 2ACTC mRNA. This confirms that the productivity of recombinant protein production can be improved by using circular mRNAs. In this study, the motif “RRACH” [32] was encoded at the 5′ of the start codon. Thus, m6A-mediated translation initiation has been shown to be an effective method of translation initiation.

Circular mRNA has also shown significant potential for the development and production of biomaterials based on large proteins with tandem repeats. Li Liu et al. designed a CmRNA encoding the spider silk proteins MaSP1 and FSLP in 2022 and tested it in *E. coli* [52]. The CmRNA contained only one unit of template sequence, but infinite translation due to the circular form of mRNA resulted in long, repetitive polypeptides larger than 110 and 90 kDa, respectively. Interestingly, the efficiency of mRNA circularization of FSLP was 36.7%, while MaSP1 had a 4-fold lower efficiency. This might be related to the size of the mRNA encoding MaSp1, which is only 156 nucleotides, while the optimal size of exon is between 300 and 500, as was reported previously by Vicens et al. in 2008 [53].

### Future Directions

To date, only a few experimental papers on recombinant protein production from circular RNAs have been published. In all studies aimed at the practical outcome—the synthesis of a specific protein from circular mRNA [22,23,51,52]—only one variant of translation initiation was used. At the same time, there are many different ways to control this process using genetic engineering methods (Table 2). Moreover, several studies have shown that different constructs for initiation have an extremely different influence on protein production [19,39,49]. As far as we know, there are no studies in which the most effective variant of translation initiation for better protein expression was selected that could then be used to solve a practical problem. At the same time, the more efficiently the protein is expressed in the target tissue, the less mRNA needs to be produced. This has a direct impact on the economic viability of the drug or biotechnological product being developed.

It should be noted that the entities considered in this review (IRES, IRES-like, TEE, CITE, R2 elements, m6A) should not be strictly distinguished from each other. Indeed, IRES, IRES-like, TEE, CITE and R2 elements are in one way or another the internal ribosome entry site. Moreover, all these sequences could be termed as translation enhancement elements according to the TEE definition. In addition, each of these elements may contain an m6A modification and IRES-like sequences that can also initiate cap-independent translation initiation. The discovery of RBP, which can interact with short sequences and initiate translation, further complicates the situation. This means that the effect of one element can be determined by a combination of different ways of cap-independent translation initiation, which should be taken into account when creating new genetic constructs.

## 4. Conclusions

Circular mRNA technology offers new opportunities for rapid and cost-effective drug or biomaterial research and development. Future studies to exploit this niche should focus on increasing knowledge on the comparative efficiency of different cap-independent initiation methods and their optimization, as they are the cornerstone for the viability of circular mRNA. Currently, there are not enough data of this kind in the public domain, which limits the use of such constructs in circular mRNA construct development. Depending on the tissue of interest, a library of constructs with different variants should be tested in the appropriate model. Proper selection of the best translation initiation variant for further development could make circular mRNAs the platform of choice for protein synthesis in a variety of applications.

## Figures and Tables

**Table 1:** The efficiency of IRESs in different cell lines.

IRES	Cell Line	Method of Comparison	Results	Source
EMCV, CVB3	HEK293, HeLa, A549, Min6	Gaussia luciferase	The efficiency of IRESs varies according to cell type; CVB3 IRES was superior in all cell types	[9]
EMCV, Poliovirus, KSHV, and HCV	HEK293; U87; Huh7; 293T	GFP	Poliovirus IRES resulted in maximal expression in HEK293	[7]
EMCV and HAV	Monkey kidney cells (BT7-H)	antibiotic resistance (bacterial chloramphenicol acetyltransferase)	EMCV IRES was more efficient than translation directed by the HAV IRES	[24]
A synthetic construct with five concatenated copies of the 9-nt Gtx IRES	mouse N2a cells	Photinus luciferase	The efficiency of the translation increases, if several copies of the 9-nt Gtx IRES are included in the construct	[25]
EMCV, c-myc, FGF-2, and HTLV-1	B16.F10, TS/A, NIH-3T3, ψCRIP, 293T, and primary cultures of human melanoma cells	Immunostaining and flow cytometry	The efficiency of translation initiation depends on the type of cells induced and the presence of other genetic elements in the vector.	[26]
Five viral (FMDV, HCV, EMCV, PV, HRV) and eight cellular IRES elements (Rbm3, NRF, Apaf-1, BIP, VCIP, AQP-4, c-myc, CAT-1)	murine fibroblast cell line (NIH 3T3), mouse embryonic fibroblasts (MEF), human hepatoma cell line (Huh 7) and human lung fibroblasts (MRC-5)	Firefly and Renilla luciferase	Vascular endothelial growth factor and type 1 collagen-inducible protein (VCIP) IRES induced the highest firefly luciferase expression rate in all tested cell lines	[27]

**Table 2:** Mechanisms of translation initiation.

Type of mRNA Translation Initiation	Mechanism of Initiation	Description of the Mechanism	Features	Applicability to circRNA Translation	References
Cap-dependent	eIF4E-dependent, with scanning	eIF4E binds the cap structure and the eIF4F complex, then the cap-binding complex recruits the 40S subunit, the initiation complex scans the mRNA until it reaches the start codon, and then the 60S ribosomal subunit joins this complex	The most common, canonical way of translation of mRNAs in higher eukaryotes	Not applicable	[11,12]
	eIF4E-dependent, scanning free	There are several variants of the scanning free mechanism: - mRNA with short 5′-UTR (leaderless mRNA) translation; - translation initiator of short 5′ UTR mediated translation; - Histone H4 translation; - Ribosome shunting	Supposed to be a non-efficient process leading to leaky scanning. At the same time, several reports show efficient and accurate translation of short 5′ UTR mRNAs that are evidently translated differently from the well-known canonical scanning mechanism.	Not applicable	[54]
	eIF4E-independent	DAP5, homolog of eIF4G, which lacks eIF4E binding, forms complexes with eIF3d	About 20% of capped mRNAs are translated this way, during physiological conditions of mTOR inhibition and eIF4E depletion	Not applicable	[55]
Cap-independent	IRES-mediated initiation	IRES interacts with the 43S pre-initiation complex by direct binding via specific structural elements formed with the RNA, indirectly via ITAFs and cellular eIFs or by homology pairing of 5′UTR mRNA motifs with 18S rRNA	The translation efficiency generally is lower than cap-dependent one, but in some cases, it was shown to be equally effective, for example in the case of EMCV	Applicable, data available	[19]
	CITE-mediated	CITEs can be located both within 5′ and 3′ UTRs and bind eIF4E and/or eIF4G subunits of eIF4F. Some CITEs can also directly bind ribosomal subunits, or the ribosomes themselves, without being dependent on elF4F	Translation efficiency of CITEs varies depending on their nature (5′ or 3′ type) and the translated sequence. 3′ CITEs were shown to be an effective substitution of cap-dependent translation	No available data, but theoretically applicable	[56,57]
	m6A-mediated	m6A modification within 5`UTR of mRNA can recruit a 40S ribosomal subunit through direct eIF3 binding	m6A-mediated translation co-exists with eIF4F-mediated translation for a great deal of transcripts, thus fully capped mRNAs can undergo m6A-mediated translation providing selectivity of mRNA translation in response to environmental and physiological conditions	Applicable, data available	[58]
	Mediated by IRES-like structures	Short hexamer sequences in endogenous circRNA are capable of translation initiation	This mechanism is less effective than viral IRESs, but each sequence less than 50 nt may contain a short IRES-like element, thus most human circRNAs might have a potential for translation. However, these structures are not conserved, hardly classified, and hardly predictable	Applicable, data available	[22,33,35]
	Rolling circle amplification translation	Translation initiation of circRNAs carrying only Kozak sequence (without 5′-cap, poly(A), IRES, stop codon) is possible. The ribosome continuously circles the circRNA molecule, which leads to the production of a long repeating peptide	CircRNAs can be efficiently translated by a rolling circle amplification mechanism in a cell-free *E. coli* translation system and in human cells	Applicable, data available	[50,59]
	R2-mediated	R2 element contains conservative structures (pseudoknots) which can be recognized by the translational machinery	Efficiency depends on the retrotransposon sequence and its structure, some of the R2 elements were shown to be 35 times more effective than HCV IRES	No available data, but theoretically applicable	[60,61]
	Mediated by *cis*-Acting Sequences and Secondary Structures in 5′ and 3′UTR	The precise mechanism is still unknown, but initiation of translation depends on both secondary structures and primary sequences within UTRs. 5′ and 3′ UTRs of uncapped RNA of *Flaviviruses* must be free and present in *cis*	This mechanism was shown for DTMUV, TMUV, DENV2, ZIKA, and JEV, but it is probably common for all *Flaviviruses*	No available data, most likely not applicable	[62]
	TEE-mediated	Mechanism is unclear	TEE-mediated initiation was described only for the vaccinia virus (VACV)	No available data	[39,40,41]

## Data Availability

Not applicable.
